# Minimal important differences for the WOMAC osteoarthritis index and the Forgotten Joint Score-12 in total knee arthroplasty patients

**DOI:** 10.1186/s12891-020-03415-x

**Published:** 2020-06-23

**Authors:** N. Holtz, D. F. Hamilton, J. M. Giesinger, B. Jost, K. Giesinger

**Affiliations:** 1 Department of Orthopaedics and Traumatology, Kantonsspital St. Gallen, Rorschacher Strasse 95, CH-9000 St. Gallen, Switzerland; 2 Department of Orthopaedics and Trauma, University of Edinburgh, Edinburgh, UK; 3 Innsbruck Institute of Patient-Centered Outcome Research (IIPCOR), Innsbruck, Austria; 4 Department of Orthopaedics and Traumatology, Kantonsspital St. Gallen, Rorschacher Strasse 95, CH-9000 St. Gallen, Switzerland

**Keywords:** Minimal important difference, Forgotten Joint Score-12, Western Ontario and McMaster universities osteoarthritis index, WOMAC, Patient-reported outcomes, Total knee arthroplasty

## Abstract

**Background:**

Total knee arthroplasty (TKA) is an effective treatment for end-stage osteoarthritis. Patient reported-outcome measures (PROMs) capture the patients’ perception of the success of an intervention. The minimal important difference (MID) is an important characteristic of the PROM, which helps to interpret results. The aim of this study was to identify the MID for the Forgotten Joint Score-12 (FJS-12) and Western Ontario and McMaster Universities (WOMAC) osteoarthritis index.

**Methods:**

Data were collected in a prospective cohort study. Patients were asked to complete the FJS-12, WOMAC osteoarthritis index and transition items evaluating change over time to determine the MID. We employed an anchor-based methodology relating score change to the response categories of the transition items using both binary logistic regression and receiver operating characteristic (ROC) analysis.

**Results:**

Data from 199 patients were analysed. Mean age was 72.3 years, 58% were women. Employing binary logistic regression the MID for the FJS-12 was 10.8 points, for the WOMAC pain score 7.5 points and for the WOMAC function score 7.2 points. ROC analyses found a MID of 13.0 points for the FJS-12, 12.5 points for WOMAC pain and 14.7 points for WOMAC function.

**Conclusion:**

We report MIDs for the FJS-12 and the WOMAC Pain and Function scales in a TKA patient cohort, which can be used to interpret meaningful differences in score. In line with previous research, we found more advanced statistical methods to result in smaller MID estimates for both scores.

**Trial registration:**

Written consent for this study was obtained from all participants and ethical approval was granted by the local ethics committee (Ethikkommission St. Gallen; EKSG 14/973; Registered 03 July 2014; http://www.sg.ch/home/gesundheit/ethikkommission.html).

## Background

Total knee arthroplasty (TKA) is well established to improve pain and function in patients suffering from end-stage osteoarthritis of the knee [1]. Outcomes of surgery can be assessed in various ways; by implant survival, image-based assessment, clinical examination or patient-reported outcome measures (PROMs). PROMs offer a view of outcome from the patient perspective and are now commonplace in both research and clinical practice. Numerous PROMs have been shown to be valid and reliable for use in TKA typically evaluating pain, function, or treatment satisfaction [2–10].

PROM outcomes are typically presented as score points (usually via a 0–100 range), but this is somewhat abstract and can be difficult to interpret. To judge the effectiveness of an intervention and interpret the difference between randomised groups in a clinical trial, a meaningful quanta of change on the score range must be contextualised to determine whether a statistically significant result is also clinically meaningful.

Determining minimal important differences (MIDs) of a score can support the decision as to clinical relevance of score differences. The MID has been defined as “the smallest change in an outcome that a patient would identify as important” [11]. A variety of methodological approaches to determine the MID for a PROM exist. The most informative are anchor-based methods that relate score differences to external criteria [12]. Frequently this is done relying on transition items to assess the degree to which an individual can perceive an improvement or deterioration between two assessment time points. The responses concerning the experienced change are then related to differences in PROM scores obtained at those two time points, thus “anchoring” the PROM scores to the degree of change perceived by the patient [13]. Previously mean score change related to the magnitude of perceived change has been analyzed [11] and receiver operator characteristics (ROC) methodology have been used to calculate MIDs. More recently however Terluin et al. [14] described a logistic regression modelling approach that is more accurate and less dependent on the correlation between PROM score and anchor responses.

The Western Ontario and McMaster Universities index (WOMAC) has been used for decades as a measure of arthroplasty success [3]. It has various estimates of MID, but none derived from a logistic regression model. Various estimates have been proposed for WOMAC MID. To determine the MID in patients with OA of the knee undergoing TKA Escobar et al. [15] performed a 1-year prospective multicentre study and reported a MID of 22 points for WOMAC pain and 24 points for WOMAC function based on ROC analysis. Terwee et al. 2010 evaluated various methods of calculating MID for the WOMAC score and reported up to 29.4 points for pain and 22.8 points for function subscores using ROC analysis. Both of these estimates are unexpectedly high as they exceed the standard deviation of the baseline score.

Previously, Terwee et al. [16] calculated the MID for WOMAC in an arthroplasty population of patients undergoing primary and revision hip and knee surgery and reported lower MIDs of 16.5 points for pain and 9.6 points for function (values rescaled to a 0–100 metric for comparability). The Forgotten Joint Score-12 is a more recent PROM, developed to evaluate joint awareness [2]. This score is now well used in knee arthroplasty cohorts and is thought to offer improved discrimination of patients with high functional levels and little pain [17–20]. To date there has only been a single anchor-based estimate of MID reported for the FJS-12 that evaluated the change between pre-operative values and 1-year outcomes [21]. Ingelsrud et al. [21] calculated a MID of 14 points for change in the FJS-12 between pre-op and 1 year following TKA for primary OA in a prospective cohort study with patient data from one Danish hospital using a regression modelling approach. Calculating the MID using a ROC methodology, they found a MID of 17 points.

MIDs have been reported to be specific for patient populations, which means that a single PROM can have different MIDs in different patient groups [22]. Further MID may change across different assessment timeframes. By measuring the change between preoperative status (very poor function) and 1-year post-arthroplasty recovery (high level of function) a large difference in outcome is evident, and the most robust estimates of the minimal score change required to offer a detectably different outcome to the patient may not be apparent.

The aim of this study was to calculate MIDs after TKA for the WOMAC osteoarthritis index and the FJS-12 using the currently most accurate predictive modelling approach and assessing change at differing post-operative time points up to 2 years after surgery, to calculate the most appropriate MID.

## Methods

### Study design, setting and participants

Between 2015 and 2018 we invited patients that had undergone unilateral TKA at the Kantonsspital St. Gallen (Switzerland) and were included in the local joint registry, to participate in this prospective study. Patients were contacted via mail up to 24 months after surgery and were asked to complete the Forgotten Joint Score-12 (FJS-12), the WOMAC Osteoarthritis Index, two questionnaires assessing change in joint awareness respectively pain and function, and a clinical data form. If the completed questionnaire was not received within a two-week interval, patients received a telephone reminder and if necessary, a new questionnaire was sent by mail. Patients were excluded from the study if they had undergone any other intervention in the meantime (any knee revision surgery or knee injection on the affected side). Written consent was obtained from the participants and ethical approval was granted by the local ethics committee (EKSG 14/973).

### Forgotten joint score (FJS-12)

The FJS-12 assesses joint awareness during activities of daily living [23]. From 12 questions with five response categories a total score is calculated ranging from 0 to 100 points. High values ​​indicate a high degree of being able to forget the joint in daily life (i.e. low joint awareness). The FJS-12 has been validated in a number of studies [2, 24–26] and has been shown to have notably low ceiling effects and a wide measurement range [27], excellent retest reliability [28, 29], and high responsiveness to change [17].

### WOMAC osteoarthritis index

Bellamy and Buchnan [7] introduced the WOMAC osteoarthritis (OA) index in 1988 as a self-reported outcome measure in patients with lower limb OA. The original score with 5-point Likert response categories consists of 24 questions covering three dimensions: pain (five questions), stiffness (two questions) and function (17 questions). Multiple studies tested the WOMAC osteoarthritis index for validity, reliability, feasibility and responsiveness to measure changes after different OA interventions [3, 16]. The WOMAC osteoarthritis index is scored from 0 to 100 points. In our study, a high score is associated with less severe impairment for ease of interpretation and comparison to the other scores.

### Transition items for assessing change in outcome

To determine the MID with an anchor-based approach we used transition items assessing the change in an outcome parameter between the study time point and a previous reference time point.

For patients completing the questionnaires up to 1 year after surgery the transition items asked about change in outcome parameters (joint awareness, pain, and function) since their preoperative status. Patients responding more than 1-year after surgery, were asked to rate changes since the 1-year follow-up visit at the hospital.

We used three transition items relating to change in joint awareness, pain and function, with seven response categories:

- How has your pain in the operated knee changed since the pre-operative/1-year follow-up visit at the hospital?

Much worse - Worse - A little worse - Neither better nor worse - A little better - Better - Much better

- How have your difficulties with your operated knee regarding daily activities changed since the pre-operative/1-year follow-up visit at the hospital?

Much worse - Worse - A little worse - Neither better nor worse - A little better - Better - Much better

- Compared to the pre-operative/1-year follow-up visit at the hospital are you more or less aware of your operated knee?

Much more - More - A little more - Neither more nor less - A little less - Less - Much less

### Statistical analyses

Patient data are described with means and standard deviations. FJS-12 and WOMAC osteoarthritis index change by anchor response categories are displayed as boxplots. The association between FJS-12 and WOMAC osteoarthritis index change and responses to the transition items was assessed with the Spearman correlation.

Change in FJS-12 and WOMAC osteoarthritis index between the assessment and the reference point (pre-surgery and 1-year follow-up respectively) was calculated as the difference between the score at the study time point and the score at the reference time point (for which FJS-12 and WOMAC data was available from the local joint registry).

Estimation of MIDs were determined anchor-based by relating change in PRO scores to the response categories of the transition items. The primary analysis was by predictive modelling [14] using a binary logistic regression model to predict the dichotomous criterion: improvement (a little better – better – much better) versus no improvement (neither better nor worse – a little worse – worse – much worse) using the change in PRO score between the time points as continuous predictor. The model did not account for repeated assessments in the same patient. In such a model the MID equals the score change that provides the same probability for belonging to the improved or the not-improved group. Additionally, we analysed a possible difference in MID for the first and second year after surgery by adding a binary timepoint variable (follow-up assessment before vs after 12 months post surgery) to the logistic regression model. This model comprised the independent variables: change in PRO score, timepoint, and their two-way interaction.

As a secondary analysis we also determined MIDs using ROC analysis that estimates the MID by relating the score change (a metric variable), to the binary variable (no improvement vs improvement) derived from the transition item. The change score provides highest sensitivity and specificity (i.e. the highest Youden’s J statistic) for predicting improvement is selected as the MID.

## Results

Of 309 patients that were eligible and were contacted for participation in the study, a total of 199 patients (64.4%) provided complete questionnaires for analysis. Mean patient age was 72.3 years (SD 9.9) and 116 (58.3%) were female. A total of 104 prostheses were implanted to the right knee (52.3%) and 95 to the left (47.7%) (Table 1). In the first period (up to 12 months, with pre-op score as reference), 162 patients provided 162 change assessments, i.e. no patient provided two change assessments. In the second period, (after 12 months, with 12-month score as reference), 37 patients provided 51 change assessments, i.e. 14 patients provided two assessments. This resulting in 213 individual study time points (with corresponding reference time points) available for assessment. Descriptive data for these time points is presented in Table 2.

**Table 1:** Sample characteristics

Age	Mean (SD)	72.3 (9.9)
BMI	Mean (SD)	30.5 (5.3)
Sex N (%)	female	116 (58.3%)
	male	83 (41.7%)
Side N (%)	Left	95 (47.7%)
	right	104 (52.3%)

**Table 2:** Descriptive statistics for FJS-12 and WOMAC osteoarthritis index across study time points

Time point	N	FJS-12 Mean (SD)	WOMAC Pain Mean (SD)	WOMAC Function Mean (SD)
Reference time point: pre-surgery^a^	162	11.7 (11.8)	47.2 (19.6)	41.7 (17.1)
Follow-up: 2 months	36	32.9 (27.5)	83.9 (14.4)	77.0 (17.2)
Follow-up: 3 months	31	41.2 (26.1)	85.6 (15.2)	76.5 (15.3)
Follow-up: 4 months	31	51.8 (24.7)	84.2 (16.0)	80.6 (14.3)
Follow-up: 5 months	24	50.7 (27.7)	90.4 (10.8)	81.8 (14.1)
Follow-up: 12 months	40	68.6 (27.1)	92.3 (10.7)	84.2 (14.1)
Reference time point: 12 months^b^	37	59.3 (31.5)	89.2 (15.6)	79.4 (19.8)
Follow-up: 15 months	12	68.8 (24.6)	94.6 (8.4)	86.8 (9.2)
Follow-up: 18 months	16	59.0 (25.6)	88.8 (15.5)	78.2 (19.4)
Follow-up: 24 months	23	54.0 (31.0)	85.4 (19.2)	77.4 (19.6)

^a^reference score for the 162 patients completing the transition item (change assessment) between 2 and 12 months after surgery

^b^reference score for the 37 patients completing the transition item (change assessment) between 15 and 24 months after surgery. In 14 patients change was assessed at two different time points (i.e. 37 patients provided 51 assessments)

**Fig. 1:**
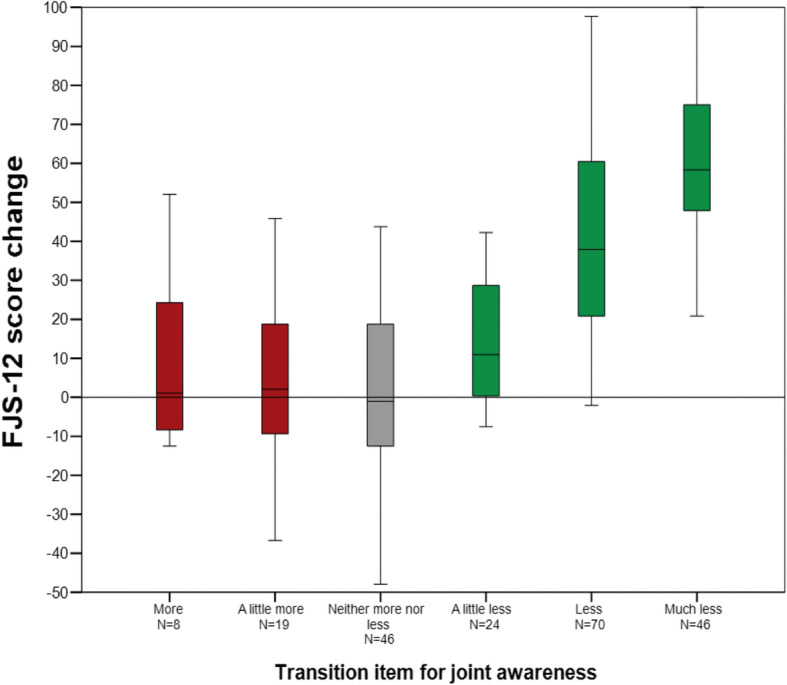
FJS-12 score change for each category of the transition item for joint awareness (please note that no patient reported that joint awareness was “much more”)

**Fig. 2:**
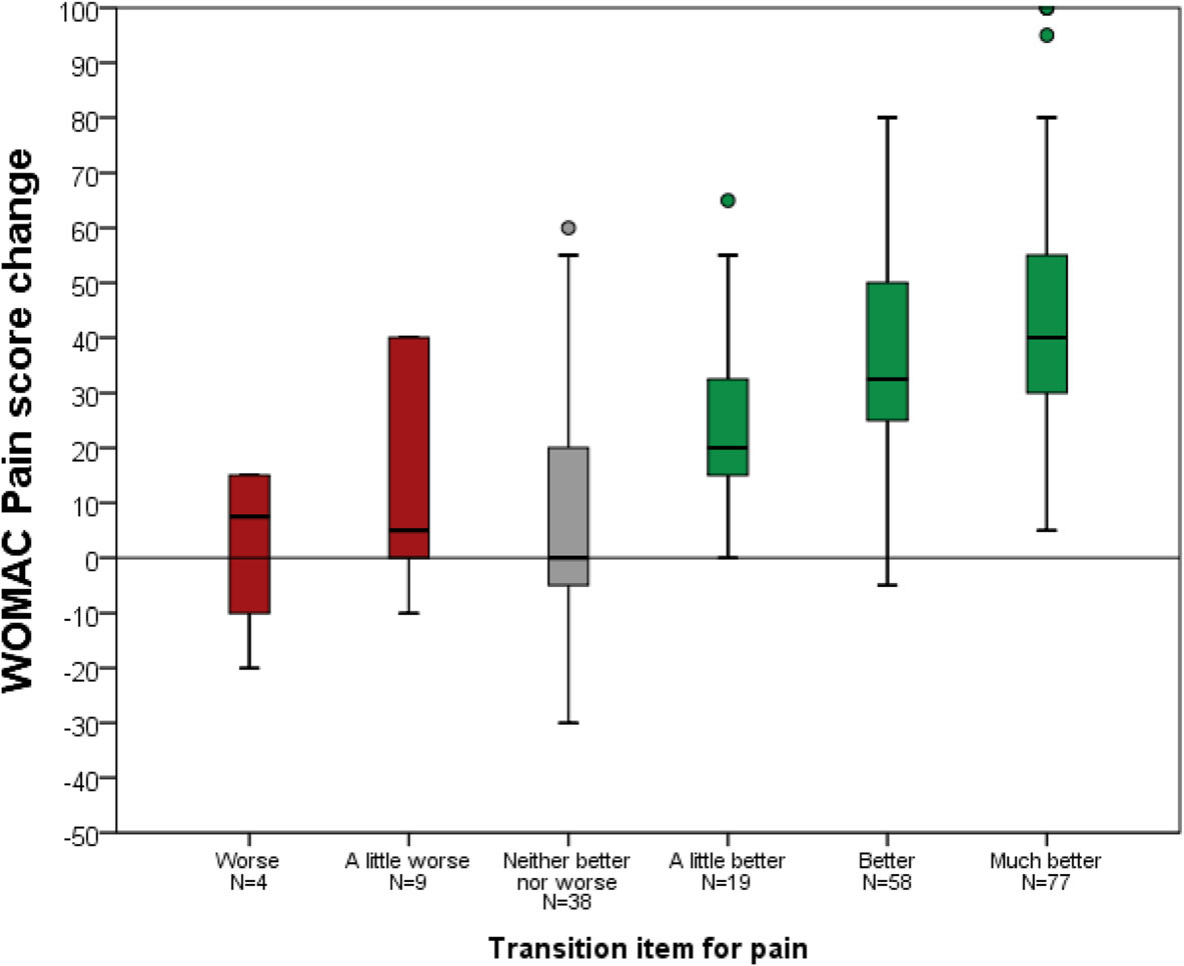
WOMAC Pain score change for each category of the transition item for pain (please note that no patient reported that pain was “much worse”)

**Fig. 3:**
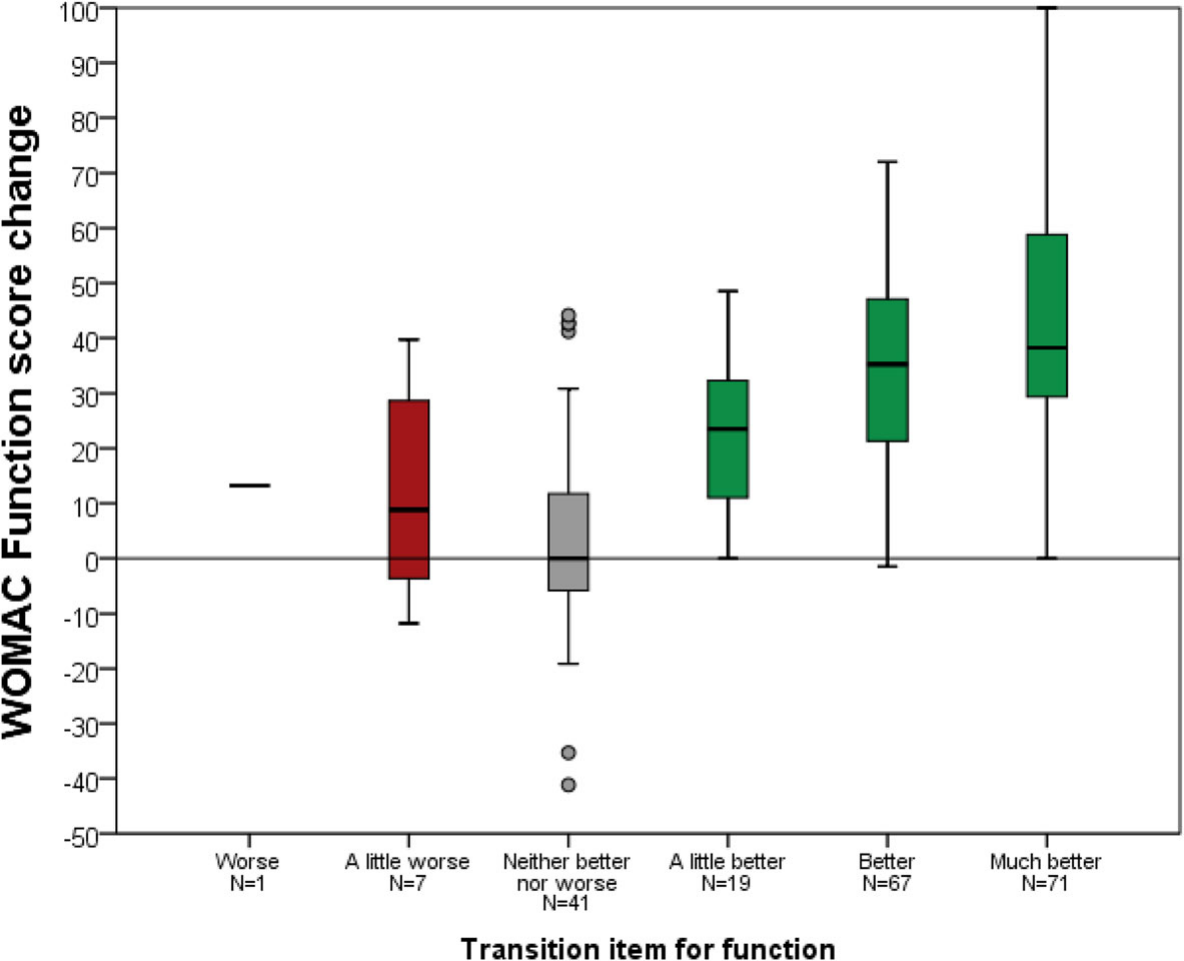
WOMAC Function score change for each category of the transition item for function (please note that no patient reported that function was “much worse”)

An improvement of joint awareness was reported by 65.7% of patients, compared to 75.1% of patients reporting less pain and 76.2% reporting better function. Details are given in Table 3. Mean score change for the FJS-12 and the WOMAC are shown for each response category of the transition items in Fig. 1, 2 and 3.

**Table 3:**
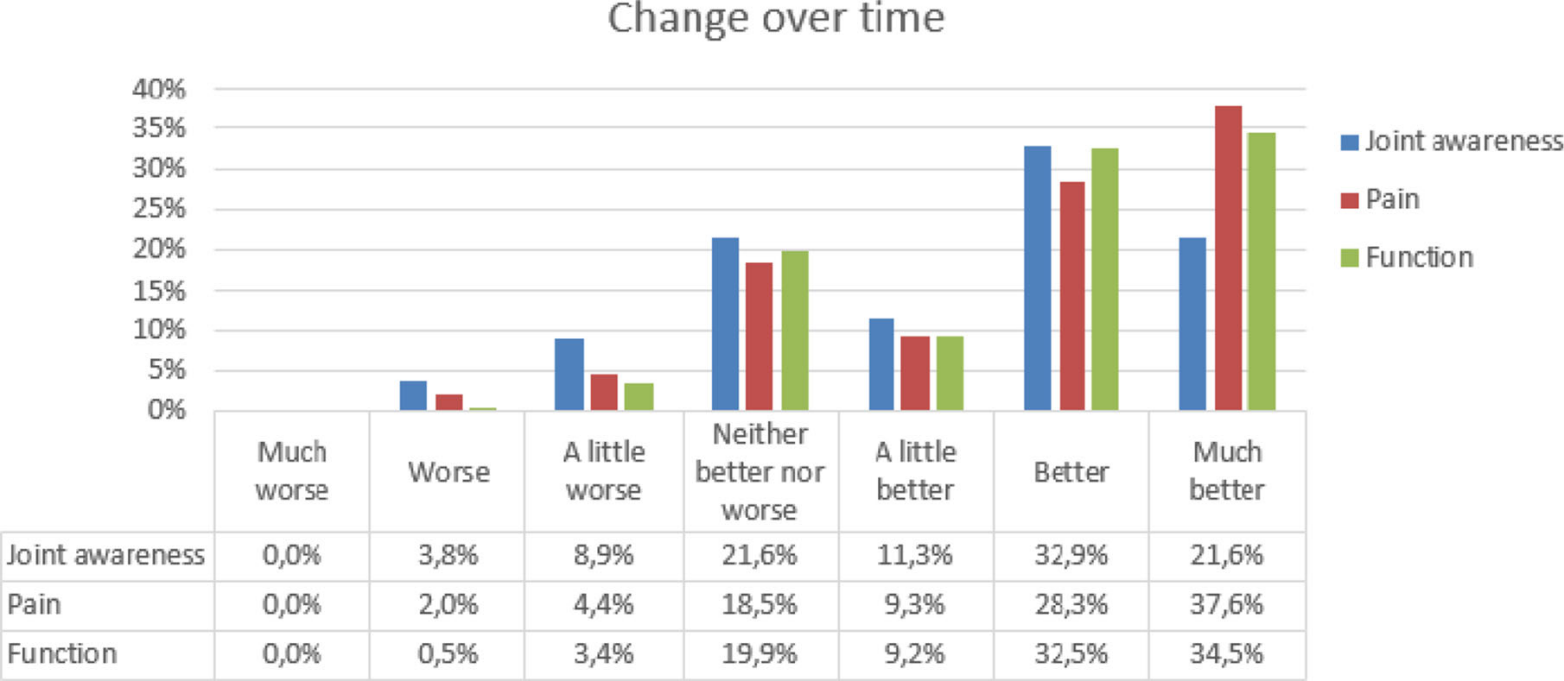
Descriptive statistics for the transition items for joint awareness (*N* = 213), pain (*N* = 205) and function (*N* = 206)

For the FJS-12 the correlation between the transition item and change between the study time point and the reference time point was *r* = 0.59 (*p* < 0.001). For WOMAC Pain this correlation was *r* = − 0.45 (*p* < 0.001) and for WOMAC Function r = − 0.48 (*p* < 0.001). Binary logistic modelling offered narrower minimal change estimates than ROC methodology, Table 4.

**Table 4:** MIDs for the FJS-12, WOMAC Pain and WOMAC Function scores

	MID derived from logistic regression analysis	MID derived from ROC analysis
FJS-12	10.8 points	13.0 points (sensitivity 0.84, specificity 0.71)
WOMAC Pain	7.5 points	12.5 points (sensitivity 0.91, specificity 0.67)
WOMAC Function	7.2 points	14.7 points (sensitivity 0.85, specificity 0.76)

Using regression modelling, the MID was 10.8 points for the FJS-12 (constant = − 0.679, beta = 0.063). For the WOMAC Pain scale (regression model: constant = − 0.514, beta = 0.069) the MID was 7.5 and for the WOMAC Function scale (regression model: constant = − 0.648, beta = 0.089) the MID was 7.2 points. Beta coefficients were statistically significant (*p* < 0.001) in all models.

In an additional analysis, we investigated a possible change of size of MID over time, but did not find a difference comparing the first and second year post-surgery for the FJS-12 (*p* = 0.983), WOMAC Pain (*p* = 0.171), and WOMAC Function (*p* = 0.910).

Using ROC analysis, a MID of 13.0 points (sensitivity 0.84 and specificity 0.71) was determined for the FJS-12. The AUC was 0.87 (95%CI 0.82–0.91), For the WOMAC Pain scale the MID from the ROC analysis was 12.5 points (sensitivity 0.91, specificity 0.67) with an AUC of 0.83 (95%CI 0.75–0.90). For WOMAC Function the MID was 14.7 points (sensitivity 0.85, specificity 0.76) with an AUC of 0.87 (0.81–0.93).

## Discussion

We have established a minimal important difference of 10.8 points for the FJS-12, 7.5 points for the WOMAC Pain scale and of 7.2 points for the WOMAC Function scale using a binary regression modelling approach. These scores reflect the minimal score improvement needed to represent a meaningful difference in patient outcomes detectable using these tools. As only a very low number of patients experienced a deterioration of their joint health, we could not calculate MIDs for deterioration based on this sample. The correlations between the transition items and the change scores indicate the validity of the change measurement.

In our study, we evaluated change across multiple different time points up to 2 years post-surgery to cover different magnitudes of change, ranging from small to large improvements. This approach differs from previous studies [15, 21, 30] that determined MIDs based on data collected within the first year after surgery that is in a time span when patients mostly experience large improvements.

We report MID values using the most recent predictive modelling techniques as this is thought to offer more accurate estimates than traditional ROC methodology [14]. As is suggested by King [31], we also report values from a different methodology, i.e. ROC analysis, which also allows comparison to wider literature. As with previous studies [15, 21, 30] we demonstrate variation in MID estimate with evaluation methodology.

The only previous estimate of MID for the FJS-12 in literature is reported by Ingelsrud et al. [21] These authors calculated a MID of 14.2 points for change in the FJS-12 between pre-op and 1 year following TKA in a Danish population using a predictive modelling approach. As we included patients up to 2 year after surgery and measured change not only in comparison to pre-surgery but also against 1-year follow-up data, we assume that the average improvement between the study time point and the reference time point was substantially smaller in our sample compared to the data presented by Ingelsrud et al.. We hypothesize that this may impact on the size of the MID, possibly as a result of patient expectations towards postoperative improvement, i.e. in the early post-operative recovery phase, when patients expect large improvements, smaller improvements may not be perceived by the patient as such. Relying on data only up to 1 year may than result in overestimation of the MID for later follow-up time points.

The WOMAC is an older tool and has undergone rigorous methodological evaluation over the years. As such, a number of studies have reported MIDs for TKA populations, therefore allowing for better comparison of our study results and statistical methods. We are not aware of any study that reports predictive modelling MID estimates for the WOMAC osteoarthritis index, however we can contrast our ROC analysis estimates. Escobar et al. reported WOMAC Pain to be 22 points (20.2–23.8 points) and WOMAC Function to be 24 points (22.7–24.7 points) using the ROC methodology. These estimates are uncomfortably high as they exceed the standard deviation of the baseline score. The review by Devji et al. [32] provides an overview over further MID estimates in non-surgical populations with values ranging from 3.6 to 7.8 for pain and 4.7 to 17.1 for function. We would like to note that the lower estimates might relate to a different WOMAC scoring, that is resulting in a metric not ranging from 0 to 100. Terwee et al. [16] reported values of 16.5 for pain and 9.6 for function (rescaled to 0–100 for comparability to our study). It is important to note that Terwee’s study included a mixed population of hip and knee patients undergoing joint arthroplasty.

Overall, the MIDs for improvement observed in our study were somewhat smaller than those determined in previous studies. A reason may be that we covered a longer follow-up period than most other studies on MIDs. We argue that studies assessing change only in the first year after surgery, when patients generally experience large improvements may overestimate the MIDs, or more specifically, may result in MIDs that are not valid for later follow-up periods. The differences between MIDs from ROC analysis and predictive modelling may reflect that MID estimates from ROC analysis are less precise [14]. While in the study by Ingelsrud et al. [21], MIDs based on ROC analysis were as well larger than MIDs from predictive modelling, this differences is substantially more pronounced in our analysis.

A limitation of our study was the size of our convenience sample that, while large enough for determining overall MIDs, did not allow comparing statistically MIDs for different follow-up periods, or specific patient subgroups.

## Conclusion

Our study has established MIDs for improvement for the FJS-12 and the WOMAC Pain and Function scores in a TKA patient cohort. In line with previous research, we found more advanced statistical methods to result in smaller MID estimates. Future research should investigate if smaller changes are of clinical importance during later follow-up periods compared to the early post-operative recovery period.

## Data Availability

The anonymised datasets used and/or analysed during the current study are available from the corresponding author on reasonable request.

## Abbreviations

FJS-12: Forgotten Joint Score-12
MIDs: Minimal important differences
OA: Osteoarthritis
PROMs: Patient-reported outcome measures
ROC: Receiver operator characteristics
TKA: Total knee arthroplasty
WOMAC: Western Ontario and McMaster Universities index
